# Supplementary material for: Eco-evolutionary Dynamics Set the Tempo and Trajectory of Metabolic Evolution in Multispecies Communities
Source: Curr Biol. 2020 Dec 21;30(24):4984–4988.e4. doi: 10.1016/j.cub.2020.09.028 (PMC7758711; doi:10.1016/j.cub.2020.09.028)
Supplement: Document S1. Figures S1–S4 [file mmc1.pdf]

Current Biology, Volume *30*

Supplemental Information

Eco-evolutionary Dynamics Set the Tempo and Trajectory of Metabolic Evolution in Multispecies Communities

Rachael Evans, Andrew P. Beckerman, Rosanna C.T. Wright, Simon McQueen-Mason, Neil C. Bruce, and Michael A. Brockhurst

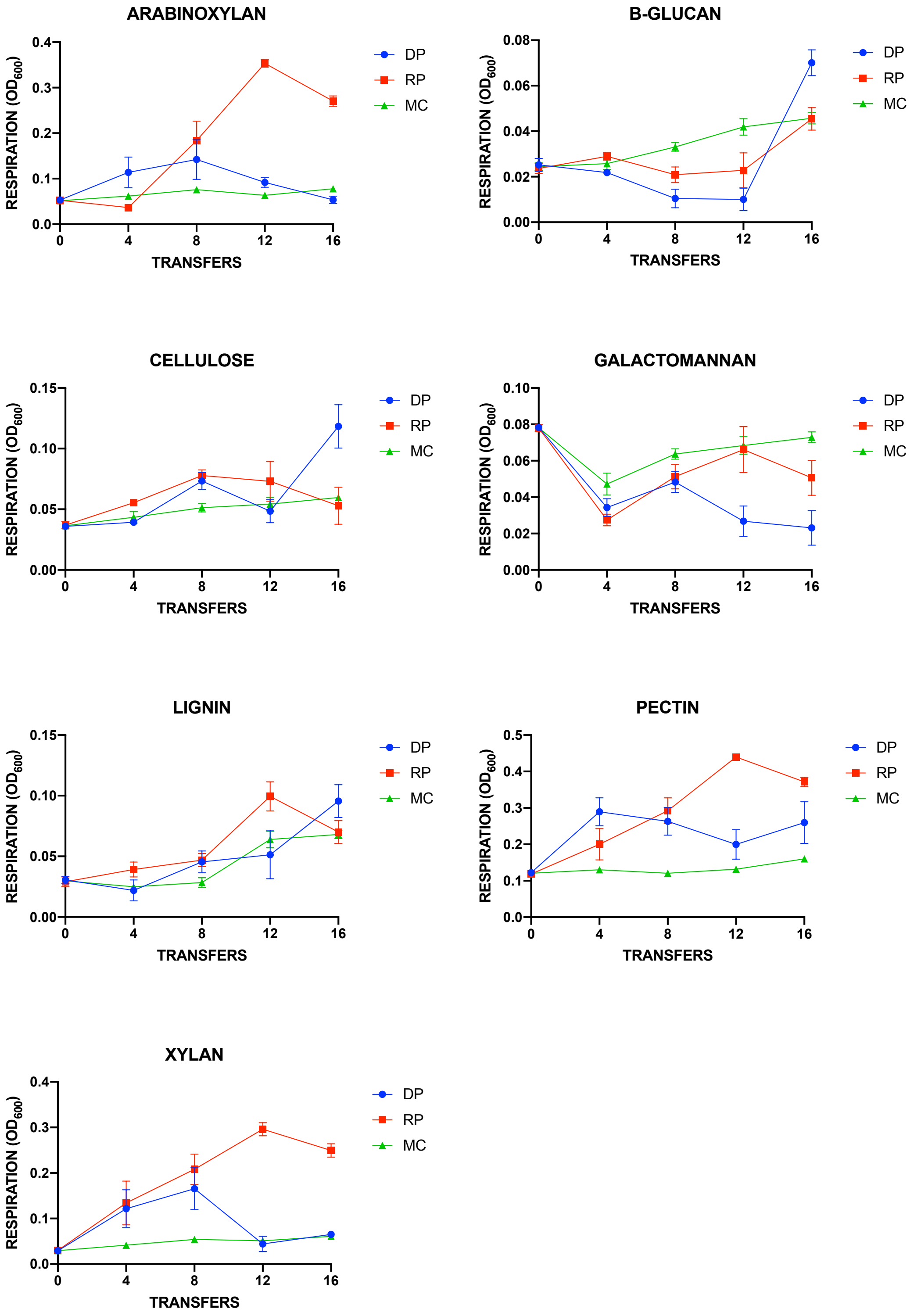

Figure S1 | Carbon substrate use over time. Related to Figure 1. Dots indicate mean ± s.e.m. respiration on a carbon substrate measured as optical density absorbance at 600 nm wavelength for *Stenotrophomonas* sp. populations from the evolution treatments [monoculture (MC; green),

reset polyculture (RP; red), dynamic polyculture (DP; blue)] over time denoted as the number of serial transfers. Panels show utilisation of different carbon substrates as indicated by the title of each panel.

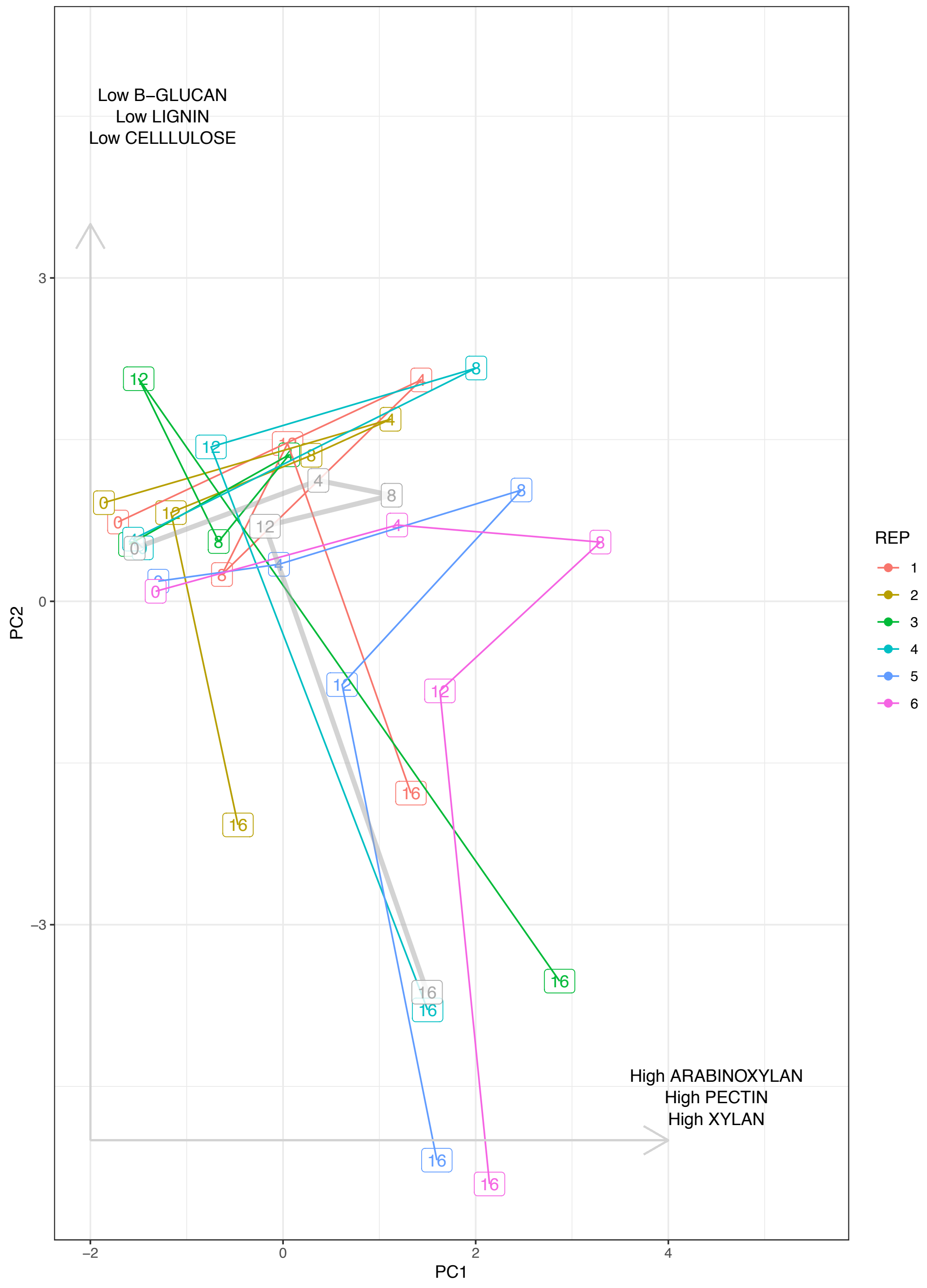

Figure S2 | Individual replicate trajectories of *Stenotrophomonas* sp. metabolic phenotype evolution in the Dynamic Polyculture treatment. Related to Figure 1. Ordination plot from a Principal Components Analysis, lines show evolutionary trajectories for the individual replicate from the dynamic polyculture treatment. Colours denote replicate as detailed in the graphical key.

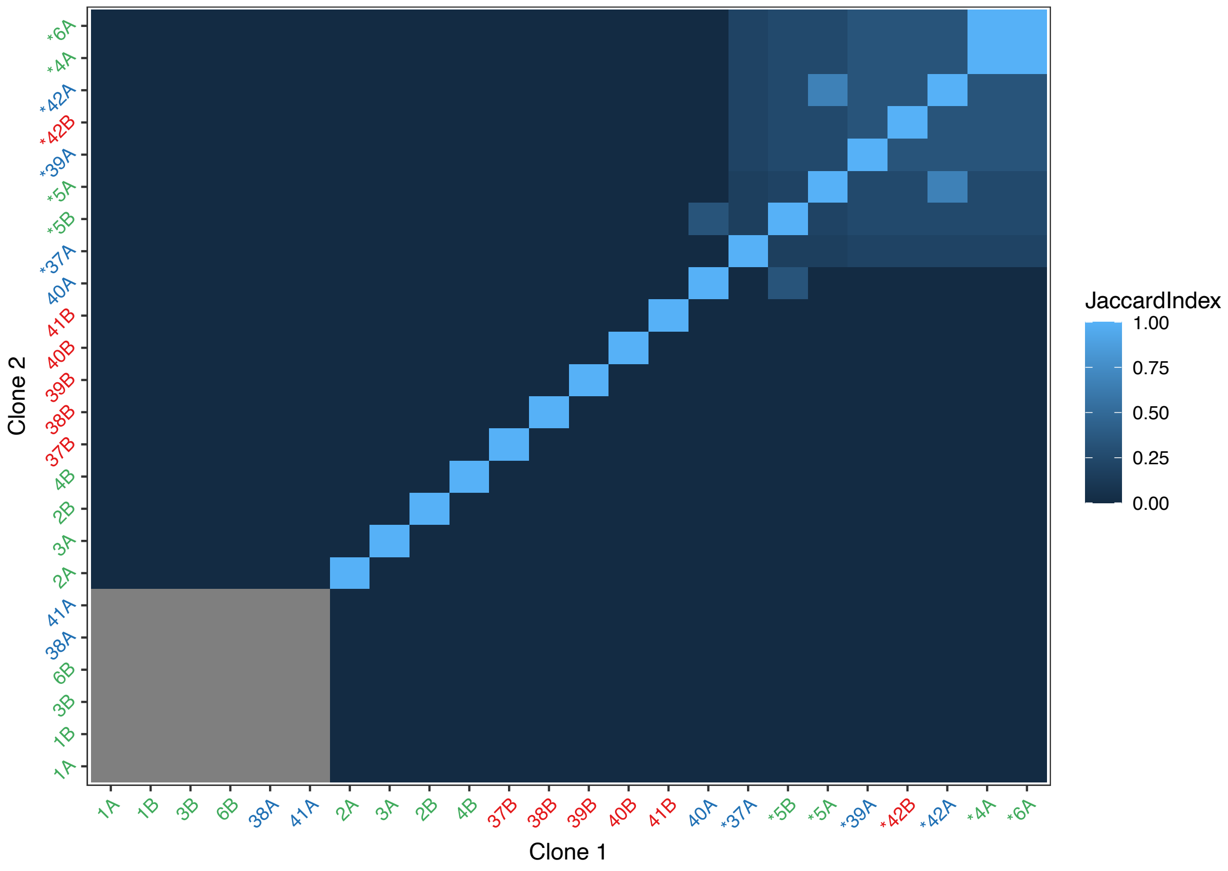

Figure S3 | Pairwise genetic similarity of all sequenced clones. Related to Figure 4. Similarity matrix showing Jaccard index values for all pairwise combinations of sequenced clones. Jaccard index was calculated as number of loci targeted in common divided by the total number of loci targeted, such that 0 (dark blue) indicates no loci in common, and 1 (light blue) indicates identical mutational profiles. Grey indicates that sequenced clones had no identifiable mutations. Clone name labels are coloured according to their treatment: green, monoculture (MC); red, reset polyculture (RP); blue, dynamic polyculture (DP). Asterisks denote clones carrying a mutation in the *tctE* gene. Each clone name relates to a circle plotted in Figure 4, as follows: from the innermost to the outermost circle in Figure 4, clones are arranged from 1A to 6A then 1B to 6B for the monoculture treatment (green), from 37A to 42A for the dynamic polyculture treatment (blue), and from 37B to 42B for the reset polyculture treatment (red).

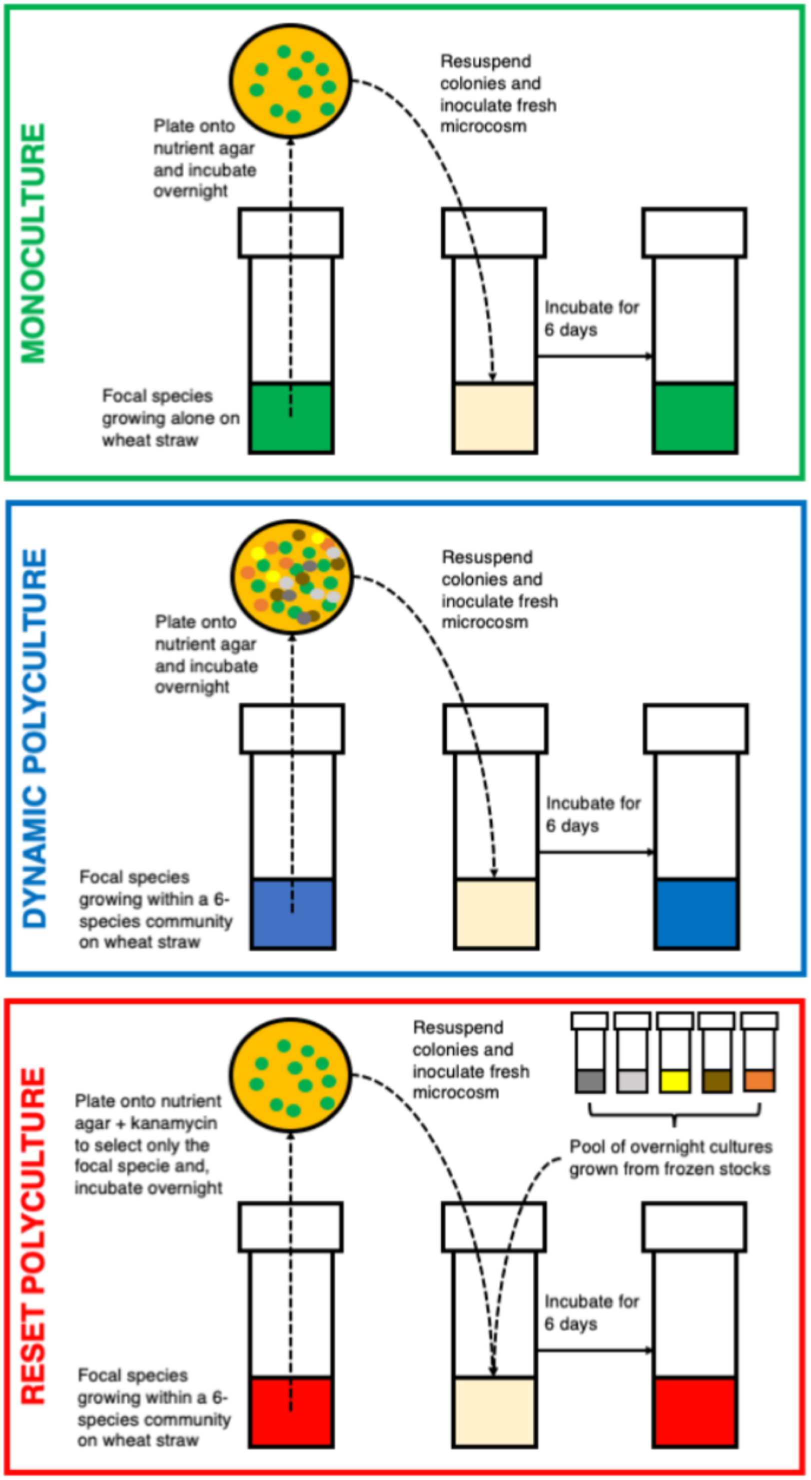

Figure S4 | Schematic diagram of the growth cycles used in each treatment. Related to the STAR Methods.
