# Supplementary material for: Eco-evolutionary Dynamics Set the Tempo and Trajectory of Metabolic Evolution in Multispecies Communities
Source: Curr Biol. 2020 Dec 21;30(24):4984–4988.e4. doi: 10.1016/j.cub.2020.09.028 (PMC7758711; doi:10.1016/j.cub.2020.09.028)
Supplement: Document S2. Article plus Supplemental Information [file mmc4.pdf]

Report

Current Biology

Eco-evolutionary Dynamics Set the Tempo and Trajectory of Metabolic Evolution in Multispecies Communities

Highlights

- Living in a multispecies community accelerated bacterial metabolic evolution
- Species sorting altered the trajectory of metabolic evolution between communities
- Eco-evolutionary dynamics drove increased use of hard-to-digest substrate niches
- This was linked to mutation of *tctE*, encoding a regulator of carbon metabolism

Authors

Rachael Evans, Andrew P. Beckerman, Rosanna C.T. Wright, Simon McQueen-Mason, Neil C. Bruce, Michael A. Brockhurst

Correspondence

michael.brockhurst@manchester.ac.uk

In Brief

Evans et al. show that living in a multispecies community accelerates metabolic evolution of a focal bacterium adapting to lignocellulose as a resource. Eco-evolutionary community dynamics prevent the focal species evolving to exploit the metabolic activity of its competitors, instead driving its adaptation to use hard-to-digest substrate niches.

Evans et al., 2020, Current Biology *30*, 4984–4988
December 21, 2020 © 2020 The Authors. Published by Elsevier Inc.
https://doi.org/10.1016/j.cub.2020.09.028

CellPress

CellPress
OPEN ACCESS

Current Biology

Report

Eco-evolutionary Dynamics Set the Tempo and Trajectory of Metabolic Evolution in Multispecies Communities

Rachael Evans,[1,2] Andrew P. Beckerman,[1] Rosanna C.T. Wright,[1,2,3] Simon McQueen-Mason,[2] Neil C. Bruce,[2] and Michael A. Brockhurst[3,4,5,*]
[1]Department of Animal and Plant Sciences, University of Sheffield, Western Bank, Sheffield S10 2TN, UK
[2]Department of Biology, University of York, Wentworth Way, York YO10 5DD, UK
[3]Division of Evolution and Genomic Sciences, University of Manchester, Dover Street, Manchester M13 9PT, UK
[4]Twitter: @brockhurstlab
[5]Lead Contact
*Correspondence: michael.brockhurst@manchester.ac.uk
https://doi.org/10.1016/j.cub.2020.09.028

SUMMARY

The eco-evolutionary dynamics of microbial communities are predicted to affect both the tempo and trajectory of evolution in constituent species [1]. While community composition determines available niche space, species sorting dynamically alters composition, changing over time the distribution of vacant niches to which species adapt [2], altering evolutionary trajectories [3, 4]. Competition for the same niche can limit evolutionary potential if population size and mutation supply are reduced [5, 6] but, alternatively, could stimulate evolutionary divergence to exploit vacant niches if character displacement results from the coevolution of competitors [7, 8]. Under more complex ecological scenarios, species can create new niches through their exploitation of complex resources, enabling others to adapt to occupy these newly formed niches [9, 10]. Disentangling the drivers of natural selection within such communities is extremely challenging, and it is thus unclear how eco-evolutionary dynamics drive the evolution of constituent taxa. We tracked the metabolic evolution of a focal species during adaptation to wheat straw as a resource both in monoculture and in polycultures wherein on-going eco-evolutionary community dynamics were either permitted or prevented. Species interactions accelerated metabolic evolution. Eco-evolutionary dynamics drove increased use of recalcitrant substrates by the focal species, whereas greater exploitation of readily digested substrate niches created by other species evolved if on-going eco-evolutionary dynamics were prevented. Increased use of recalcitrant substrates was associated with parallel evolution of *tctE*, encoding a carbon metabolism regulator. Species interactions and species sorting set, respectively, the tempo and trajectory of evolutionary divergence among communities, selecting distinct ecological functions in otherwise equivalent ecosystems.

RESULTS AND DISCUSSION

Rapid evolution of constituent species has been observed across a range of experimental [8–16] and natural [17–20] microbial communities, including within the human microbiome [21, 22], with implications for understanding how these communities are structured [23] and how they function [24, 25]. Disentangling the drivers of natural selection within such communities is extremely challenging but is essential to enable the manipulation of microbial communities for improved function [24–27]. Here, to understand the contribution of eco-evolutionary community dynamics to natural selection, we track metabolic evolution by *Stenotrophomonas* sp. during adaptation to wheat straw with or without a community of five additional, naturally co-occurring species previously isolated from compost [28]. Wheat straw is a complex carbon source comprised of secondary plant cell-walls composed of cellulose, hemicellulose, and lignin. This lignocellulosic biomass is difficult to digest, as the cellulose exists as crystalline microfibrils, the hemicellulose is a complex, highly branched and crosslinked polymer, and these polysaccharides are sealed in lignin, a complex polyphenol [29]. Nevertheless, microbial communities efficiently degrade lignocellulose across a range of natural environments including animal digestive tracts and soils [30]. Replicate microcosms were serially transferred for sixteen 7-day growth-cycles both in monoculture (MC; n = 12) and in six-species polycultures where either the eco-evolutionary dynamics were reset at each serial transfer (long-term change in the relative abundance of taxa—i.e., species sorting—and evolution of other community members not permitted = reset polyculture; RP; n = 6) or allowed to play out (species sorting and evolution of other community members permitted = dynamic polyculture; DP; n = 6). This equated to approximately 125 generations or 150 generations of *Stenotrophomonas* evolution in monoculture and polyculture, respectively, due to slightly higher growth rates in polycultures. Metabolic evolution by *Stenotrophomonas* sp. was measured as respiration by evolved

 © 2020 The Authors. Published by Elsevier Inc.
This is an open access article under the CC BY license (http://creativecommons.org/licenses/by/4.0/).

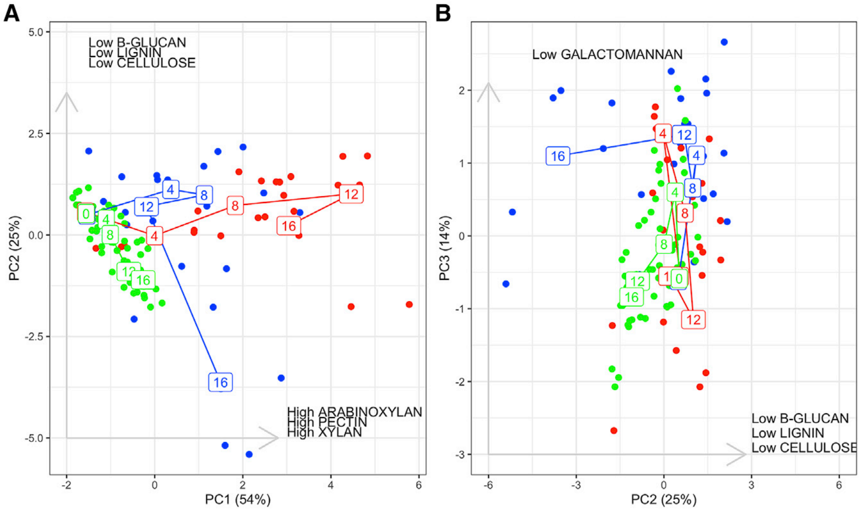

Figure 1. Trajectories of *Stenotrophomonas* sp. Metabolic Phenotype Evolution

Ordination plots from a principal components analysis (PCA) of the *Stenotrophomonas* sp. metabolic phenotype over time. The first 3 principal components (PC) captured 92% of the variation in substrate use, and thus, these PCs were plotted to visualize the evolutionary trajectories of our treatments. Plots show (A) PC1 (54%) against PC2 (25%) and (B) PC2 against PC3 (14%). The variation in resource use associated with each PC is stated on each axis. Lines show evolutionary trajectories for the *Stenotrophomonas* sp. metabolic phenotype in the monoculture (MC; green), reset polyculture (RP; red), and dynamic polyculture (DP; blue) treatments; dots show values for each individual replicate over time (denoted by transfer number labels on each line). Use of individual substrates over time are plotted in Figure S1. Individual replicate trajectories for the DP treatment are plotted in Figure S2. The raw data is provided in Data S1.

populations on seven components of lignocellulose [28], including both harder-to-digest recalcitrant substrates (β-glucan, cellulose, lignin) and easier-to-digest labile substrates that are protected from saccharification by the structure of lignocellulose prior to its digestion (xylan, arabinoxylan, galactomannan, pectin) [29].

Resource use by *Stenotrophomonas* sp. significantly diverged between treatments over time (Figure S1; linear mixed model, treatment × substrate × time interaction, $F_{12,777} = 7.8661$, $p < 2.2 \times 10^{-16}$). We used phenotypic trajectory analysis [31] to calculate three properties—length, direction, and shape—of the evolutionary paths taken within this 7-dimensional metabolic phenotype space by our treatments. Evolutionary paths varied significantly between treatments (Figure 1; permutational manova, treatment × time interaction, F = 8.97, p < 0.001). Interspecies interactions accelerated *Stenotrophomonas* sp. metabolic phenotype evolution, as indicated by a shorter evolutionary path in the MC treatment compared to both polyculture treatments (pairwise absolute differences in path distance, RP:MC Z = 7.04, p = 0.001, DP:MC Z = 8.22, p = 0.001), which themselves evolved similar distances (RP:DP Z = 0.27, p = 0.514). The RP treatment took an evolutionary trajectory whose direction was distinct from either the MC or DP treatments (pairwise differences in path angle, RP:DP Z = 2.028, p = 0.039; RP:MC Z = 1.88, p = 0.034). While the evolutionary trajectory of the DP treatment changed direction more over time than either the MC or RP treatment trajectories (pairwise differences in path shape, DP:MC Z = 2.80, p = 0.005; RP:DP Z = 3.58, p = 0.001; RP:MC Z = 1.03, p = 0.149). This is most clearly shown by the change in direction of the DP trajectory from traversing PC1 to traversing PC2 at around transfer 12 (Figure 1A). Overall, whereas the RP treatment evolved to increase use of labile substrates (Figure 1, along PC1: xylan, arabinoxylan, and pectin), the DP treatment evolved to increase use of recalcitrant substrates (Figure 1, along PC2: β-glucan, cellulose, and lignin).

Specialization on labile substrates would allow greater exploitation of the lignocellulose digestion of competing species. Consistent with this, while being in a community always increased the growth of *Stenotrophomonas* sp. relative to its growth alone, only the RP-evolved *Stenotrophomonas* sp. populations increased their competitiveness relative to the ancestor against the ancestral polyculture community on wheat straw (Figure 2; two-way anova, treatment × growth-condition interaction, $F_{3,40} = 12.47$, $p < 0.0001$; pairwise comparison of RP versus ancestor growth in polyculture, p < 0.001). Moreover, in competition against the ancestral polyculture community, the RP-evolved *Stenotrophomonas* sp. reached a higher final relative abundance than its ancestor or the evolved *Stenotrophomonas* sp. from the DP or MC treatments (one-way anova, $F_{3,20} = 22.05$ $p < 0.0001$; pairwise Tukey tests against RP, all p < 0.001). This suggests that the on-going eco-evolutionary dynamics of communities limited the evolution of exploitative metabolic strategies by the focal species. Notably, none of the evolved *Stenotrophomonas* sp. populations showed faster growth on wheat straw alone than their ancestor (pairwise comparisons of MC, DP, and RP growth rates versus the ancestor all p > 0.05). This suggests that our growth assay may not have been sufficiently sensitive to detect differences in autonomous growth rate. It is probable that direct competition of evolved populations against their ancestor would have been more discriminating as this is the gold standard method for estimating relative fitness. However, we lacked an isogenic labeled strain of this *Stenotrophomonas* sp. environmental isolate, precluding the use of this superior method.

Differences in composition among replicate DP communities emerged over time through species sorting (Figure 3). We observed strengthening covariance of *Stenotrophomonas* sp. evolved metabolism with community structure over time (community dissimilarity × time interaction; $F_{1,87} = 6.4$, $p < 0.05$), suggesting that changes in composition selected for different evolved metabolic functions among communities. Higher recalcitrant substrate use by *Stenotrophomonas* sp. was associated with communities that had higher final relative abundance of *Bacillus* sp. (linear regression, cellulose $R^2 = 0.8625$, $F_{1,4} = 19.06$, $p = 0.012$; lignin $R^2 = 0.8625$, $F_{1,4} = 19.53$, $p = 0.0115$). Moreover, the reinvasion of three of the replicate DP communities by *Bacillus* sp. from low density (Figure 3) coincided with the change in direction of the evolutionary path of *Stenotrophomonas* sp. toward recalcitrant substrates (from PC2 to PC1: Figures 1 and S2). We previously

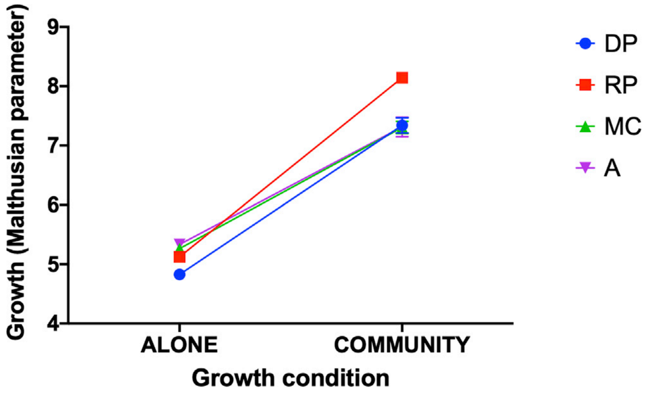

Figure 2. Growth Rates of Ancestral and Evolved *Stenotrophomonas* sp.

Growth on wheat straw when cultured alone or alongside the ancestral polyculture. Dots indicate mean growth rate ± standard error for each of the evolution treatments (monoculture [MC; green triangles], reset polyculture [RP; red squares], dynamic polyculture [DP; blue circles]) and the ancestor (purple triangles) and lines connect values measured while grown alone versus alongside the ancestral polyculture. The raw data is provided in Data S1.

showed that this *Bacillus* sp. strain is a labile substrate specialist [28], suggesting it would have competed strongly for labile substrates, potentially driving the observed niche differentiation by *Stenotrophomonas* sp. toward recalcitrant substrate use.

To examine the genetic basis of *Stenotrophomonas* sp. metabolic evolution, we genome sequenced one randomly chosen clone per replicate population. Evolved clones had acquired between 0 and 4 mutations per clone, with 33 mutations in total, of which 66.6% were non-synonymous. While treatments did not vary in the number of mutations per clone (all mutations: Welch's anova, $F_{2,10.97} = 0.03154$, $p = 0.9690$; non-synonymous mutations: Welch's anova, $F_{2,12.3} = 0.8878$, $p = 0.4363$), the genetic loci affected by non-synonymous mutations varied among treatments (Figure 4; permutational anova, $F_{5,570} = 6.304$, $p = 0.0002$). Specifically, the DP and MC treatments became significantly genetically differentiated from the RP treatment (RP:MC $t = -3.058$ $p = 0.002$; RP:DP $t = -2.216$ $p = 0.027$), but not from one another (MC:DP $t = -1.448$ $p = 0.148$). This pattern was principally driven by parallel mutation of *tctE*, which was mutated in multiple replicates of the DP (3/6 clones) and MC (4/12 clones) treatments, but in only one replicate of the RP treatment (Figure 4; Figure S3). TctD/TctE form a two-component signaling system that positively regulates tricarboxylic acid uptake in a range of species [32–35]. Furthermore, deletion of *tctD/tctE* has been shown to cause dysregulation of other signaling systems and altered expression of metabolic genes, leading to substantial alteration of carbon metabolism in *P. aeruginosa* [35]. It is probable, therefore, that *tctE* mutations played a role in the evolution of altered substrate use by *Stenotrophomonas* sp. The higher frequency of *tctE* mutations in DP-evolved compared to RP-evolved clones suggests that these mutations could be linked to the observed increase in the use of recalcitrant substrates by DP-evolved *Stenotrophomonas* populations (Figure 1, Figure S1). However, caution is required in making such inferences, in part because the single clones sequenced per population are unlikely to represent all of the genetic diversity present in the population samples used in the resource use assays.

Rapid evolutionary dynamics of constituent taxa are a feature of both experimental [8–16] and natural [17–22] microbial communities and are likely to affect the structure [23] and function of microbiomes [24, 25]. Unlike previous studies of evolution in multispecies bacterial communities [8–11, 13–15], by resetting community dynamics, we disentangled the effects of interspecies interactions from their eco-evolutionary dynamics upon the evolution of focal species' metabolism. Our data show that the evolutionary paths taken by constituent taxa depend upon the eco-evolutionary dynamics of communities. Species interactions and species sorting set, respectively, the tempo and trajectory of evolutionary divergence for focal taxa among

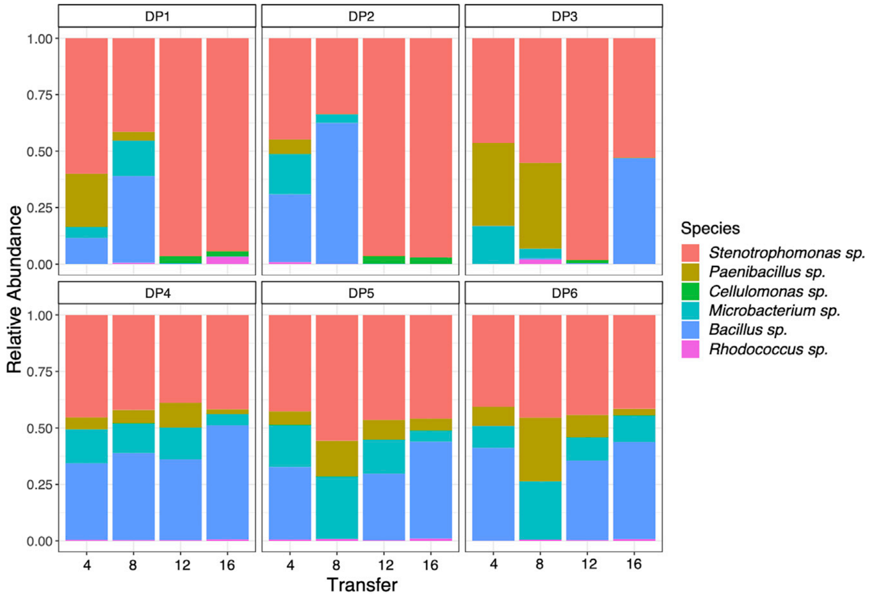

Figure 3. Relative Abundance of Species in Dynamic Polyculture Communities

Stacked bars show the relative abundance of species over time in the replicate communities (DP1 to DP6) from the dynamic polyculture treatment. The identity of species is indicated by colors as shown in the graphical key. The raw data is provided in Data S1.

CellPress
OPEN ACCESS

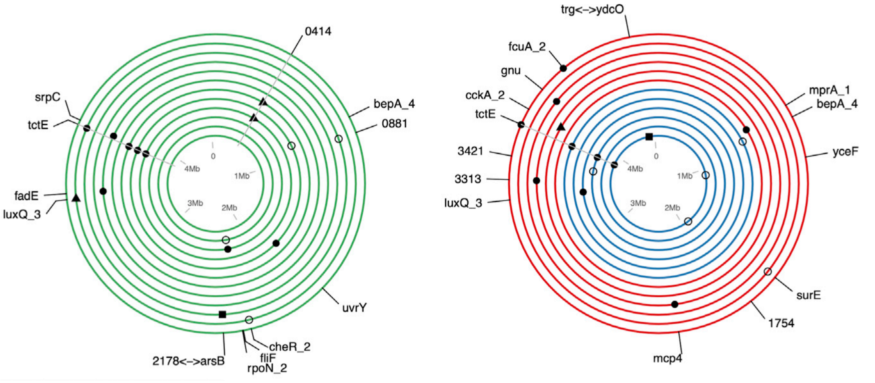

Figure 4. Parallel Genomic Evolution within and between Treatments

Circles represent the *Stenotrophomonas* sp. Genome; each concentric circle is an independent evolved clone sampled at the end of the experiment. Colors denote the monoculture (MC; green), reset polyculture (RP; red), and dynamic polyculture (DP; blue) treatments. Markers indicate genetic loci where mutations were observed in evolved clones and labels show the predicted functional annotation for these loci where available. The shape of the marker denotes the type of mutation observed: filled circle, non-synonymous SNP; open circle, synonymous SNP; triangle, insertion or deletion; square, intergenic SNP. Markers for parallel evolving loci are connected by a gray line. Figure S3 shows the pairwise genetic similarity among all sequenced clones. A complete table of sequence variants is provided in Data S2.

communities. Selection arising from the eco-evolutionary dynamics of communities can override habitat-specific adaptation [18] to select for distinct ecological functions in otherwise equivalent ecosystems. Moreover, by constraining the evolution of exploitative strategies, the eco-evolutionary dynamics of microbial communities may help to explain the stability of ecological functions that, like lignocellulose metabolism, require the collective action of multiple species in microbiomes [24, 25].

STAR★METHODS

Detailed methods are provided in the online version of this paper and include the following:

- KEY RESOURCES TABLE
- RESOURCE AVAILABILITY
  - Lead Contact
  - Materials availability
  - Data availability
- EXPERIMENTAL MODEL AND SUBJECT DETAILS
  - Bacterial strains and culture conditions
- METHOD DETAILS
  - Selection experiment
  - Resource use assays
  - Autonomous and competitive growth assays
  - Genome sequencing
  - Amplicon sequencing
- QUANTIFICATION AND STATISTICAL ANALYSIS
  - Statistical analysis
  - Genome sequencing bioinformatics
  - Amplicon sequencing bioinformatics

SUPPLEMENTAL INFORMATION

Supplemental Information can be found online at https://doi.org/10.1016/j.cub.2020.09.028.

ACKNOWLEDGMENTS

We are grateful to Helen Hipperson for her assistance with the PacBio sequencing and bioinformatics. We thank J.P.J. Hall, K. Coyte, E. Harrison, and S. O'Brien for comments on previous drafts of the manuscript. This work was funded by a BBSRC White Rose DTP PhD studentship awarded to R.E. and supervised by M.A.B., S.M.-M., and N.C.B. (BB/M011151/1), a Philip Leverhulme Prize from the Leverhulme Trust awarded to M.A.B. (PLP-2014-242), and a NERC standard grant awarded to M.A.B. (NE/R008825/1).

AUTHOR CONTRIBUTIONS

M.A.B., N.C.B., and S.M.-M. conceived the project and supervised the research; R.E. and M.A.B. designed the experiments; R.E. performed the experiments; R.E., M.A.B., A.P.B., and R.C.W. analyzed and plotted the data; M.A.B., R.E., and A.P.B. wrote the manuscript; all authors commented on the manuscript.

DECLARATION OF INTERESTS

Authors declare no competing interests.

Received: March 12, 2020
Revised: July 30, 2020
Accepted: September 4, 2020
Published: October 8, 2020

REFERENCES

1. Barraclough, T.G. (2015). How Do Species Interactions Affect Evolutionary Dynamics Across Whole Communities? Annu. Rev. Ecol. Evol. Syst. *46*, 25–48.
2. de Mazancourt, C., Johnson, E., and Barraclough, T.G. (2008). Biodiversity inhibits species' evolutionary responses to changing environments. Ecol. Lett. *11*, 380–388.
3. Fukami, T., Beaumont, H.J., Zhang, X.X., and Rainey, P.B. (2007). Immigration history controls diversification in experimental adaptive radiation. Nature *446*, 436–439.
4. Brockhurst, M.A., Colegrave, N., Hodgson, D.J., and Buckling, A. (2007). Niche occupation limits adaptive radiation in experimental microcosms. PLoS ONE *2*, e193.
5. Lanfear, R., Kokko, H., and Eyre-Walker, A. (2014). Population size and the rate of evolution. Trends Ecol. Evol. *29*, 33–41.
6. Collins, S. (2011). Competition limits adaptation and productivity in a photosynthetic alga at elevated CO2. Proc. Biol. Sci. *278*, 247–255.
7. Rainey, P.B., and Travisano, M. (1998). Adaptive radiation in a heterogeneous environment. Nature *394*, 69–72.
8. Scheuerl, T., Hopkins, M., Nowell, R.W., Rivett, D.W., Barraclough, T.G., and Bell, T. (2020). Bacterial adaptation is constrained in complex communities. Nat. Commun. *11*, 754.

9. Lawrence, D., Fiegna, F., Behrends, V., Bundy, J.G., Phillimore, A.B., Bell, T., and Barraclough, T.G. (2012). Species interactions alter evolutionary responses to a novel environment. PLoS Biol. *10*, e1001330.

10. Harcombe, W.R., Chacón, J.M., Adamowicz, E.M., Chubiz, L.M., and Marx, C.J. (2018). Evolution of bidirectional costly mutualism from byproduct consumption. Proc. Natl. Acad. Sci. USA *115*, 12000–12004.

11. Hansen, S.K., Rainey, P.B., Haagensen, J.A., and Molin, S. (2007). Evolution of species interactions in a biofilm community. Nature *445*, 533–536.

12. Paterson, S., Vogwill, T., Buckling, A., Benmayor, R., Spiers, A.J., Thomson, N.R., Quail, M., Smith, F., Walker, D., Libberton, B., et al. (2010). Antagonistic coevolution accelerates molecular evolution. Nature *464*, 275–278.

13. Zhang, Q.G., Ellis, R.J., and Godfray, H.C. (2012). The effect of a competitor on a model adaptive radiation. Evolution *66*, 1985–1990.

14. Hall, J.P.J., Harrison, E., and Brockhurst, M.A. (2018). Competitive species interactions constrain abiotic adaptation in a bacterial soil community. Evol Lett *2*, 580–589.

15. Hillesland, K.L., and Stahl, D.A. (2010). Rapid evolution of stability and productivity at the origin of a microbial mutualism. Proc. Natl. Acad. Sci. USA *107*, 2124–2129.

16. Betts, A., Gray, C., Zelek, M., MacLean, R.C., and King, K.C. (2018). High parasite diversity accelerates host adaptation and diversification. Science *360*, 907–911.

17. Koskella, B. (2013). Phage-mediated selection on microbiota of a long-lived host. Curr. Biol. *23*, 1256–1260.

18. Shapiro, B.J., Friedman, J., Cordero, O.X., Preheim, S.P., Timberlake, S.C., Szabó, G., Polz, M.F., and Alm, E.J. (2012). Population genomics of early events in the ecological differentiation of bacteria. Science *336*, 48–51.

19. Rosen, M.J., Davison, M., Bhaya, D., and Fisher, D.S. (2015). Microbial diversity. Fine-scale diversity and extensive recombination in a quasisexual bacterial population occupying a broad niche. Science *348*, 1019–1023.

20. Denef, V.J., and Banfield, J.F. (2012). In situ evolutionary rate measurements show ecological success of recently emerged bacterial hybrids. Science *336*, 462–466.

21. Zhao, S., Lieberman, T.D., Poyet, M., Kauffman, K.M., Gibbons, S.M., Groussin, M., Xavier, R.J., and Alm, E.J. (2019). Adaptive Evolution within Gut Microbiomes of Healthy People. Cell Host Microbe *25*, 656–667.e8.

22. Garud, N.R., Good, B.H., Hallatschek, O., and Pollard, K.S. (2019). Evolutionary dynamics of bacteria in the gut microbiome within and across hosts. PLoS Biol. *17*, e3000102.

23. Gómez, P., Paterson, S., De Meester, L., Liu, X., Lenzi, L., Sharma, M.D., McElroy, K., and Buckling, A. (2016). Local adaptation of a bacterium is as important as its presence in structuring a natural microbial community. Nat. Commun. *7*, 12453.

24. Foster, K.R., Schluter, J., Coyte, K.Z., and Rakoff-Nahoum, S. (2017). The evolution of the host microbiome as an ecosystem on a leash. Nature *548*, 43–51.

25. Koskella, B., Hall, L.J., and Metcalf, C.J.E. (2017). The microbiome beyond the horizon of ecological and evolutionary theory. Nat. Ecol. Evol. *1*, 1606–1615.

26. Johns, N.I., Blazejewski, T., Gomes, A.L., and Wang, H.H. (2016). Principles for designing synthetic microbial communities. Curr. Opin. Microbiol. *31*, 146–153.

27. Grosskopf, T., and Soyer, O.S. (2014). Synthetic microbial communities. Curr. Opin. Microbiol. *18*, 72–77.

28. Evans, R., Alessi, A.M., Bird, S., McQueen-Mason, S.J., Bruce, N.C., and Brockhurst, M.A. (2017). Defining the functional traits that drive bacterial decomposer community productivity. ISME J. *11*, 1680–1687.

29. Marriott, P.E., Gómez, L.D., and McQueen-Mason, S.J. (2016). Unlocking the potential of lignocellulosic biomass through plant science. New Phytol. *209*, 1366–1381.

30. Cragg, S.M., Beckham, G.T., Bruce, N.C., Bugg, T.D., Distel, D.L., Dupree, P., Etxabe, A.G., Goodell, B.S., Jellison, J., McGeehan, J.E., et al. (2015). Lignocellulose degradation mechanisms across the Tree of Life. Curr. Opin. Chem. Biol. *29*, 108–119.

31. Adams, D.C., and Collyer, M.L. (2009). A general framework for the analysis of phenotypic trajectories in evolutionary studies. Evolution *63*, 1143–1154.

32. Widenhorn, K.A., Somers, J.M., and Kay, W.W. (1989). Genetic regulation of the tricarboxylate transport operon (tctI) of Salmonella typhimurium. J. Bacteriol. *171*, 4436–4441.

33. Somers, J.M., and Kay, W.W. (1983). Genetic fine structure of the tricarboxylate transport (tct) locus of Salmonella typhimurium. Mol. Gen. Genet. *190*, 20–26.

34. Tamir-Ariel, D., Rosenberg, T., and Burdman, S. (2011). The Xanthomonas campestris pv. vesicatoria citH gene is expressed early in the infection process of tomato and is positively regulated by the TctDE two-component regulatory system. Mol. Plant Pathol. *12*, 57–71.

35. Taylor, P.K., Zhang, L., and Mah, T.F. (2019). Loss of the Two-Component System TctD-TctE in *Pseudomonas aeruginosa* Affects Biofilm Formation and Aminoglycoside Susceptibility in Response to Citric Acid. MSphere *4*, e00102-19.

36. Caporaso, J.G., Kuczynski, J., Stombaugh, J., Bittinger, K., Bushman, F.D., Costello, E.K., Fierer, N., Peña, A.G., Goodrich, J.K., Gordon, J.I., et al. (2010). QIIME allows analysis of high-throughput community sequencing data. Nat. Methods *7*, 335–336.

37. Koren, S., Walenz, B.P., Berlin, K., Miller, J.R., Bergman, N.H., and Phillippy, A.M. (2017). Canu: scalable and accurate long-read assembly via adaptive *k*-mer weighting and repeat separation. Genome Res. *27*, 722–736.

38. Hunt, M., Silva, N.D., Otto, T.D., Parkhill, J., Keane, J.A., and Harris, S.R. (2015). Circlator: automated circularization of genome assemblies using long sequencing reads. Genome Biol. *16*, 294.

39. Walker, B.J., Abeel, T., Shea, T., Priest, M., Abouelliel, A., Sakthikumar, S., Cuomo, C.A., Zeng, Q., Wortman, J., Young, S.K., and Earl, A.M. (2014). Pilon: an integrated tool for comprehensive microbial variant detection and genome assembly improvement. PLoS ONE *9*, e112963.

40. Seemann, T. (2014). Prokka: rapid prokaryotic genome annotation. Bioinformatics *30*, 2068–2069.

41. Moriya, Y., Itoh, M., Okuda, S., Yoshizawa, A.C., and Kanehisa, M. (2007). KAAS: an automatic genome annotation and pathway reconstruction server. Nucleic Acids Res. *35*, W182-5.

42. Apweiler, R., Attwood, T.K., Bairoch, A., Bateman, A., Birney, E., Biswas, M., Bucher, P., Cerutti, L., Corpet, F., Croning, M.D.R., et al. (2001). The InterPro database, an integrated documentation resource for protein families, domains and functional sites. Nucleic Acids Res. *29*, 37–40.

43. Li, H., and Durbin, R. (2009). Fast and accurate short read alignment with Burrows-Wheeler transform. Bioinformatics *25*, 1754–1760.

44. McKenna, A., Hanna, M., Banks, E., Sivachenko, A., Cibulskis, K., Kernytsky, A., Garimella, K., Altshuler, D., Gabriel, S., Daly, M., and DePristo, M.A. (2010). The Genome Analysis Toolkit: a MapReduce framework for analyzing next-generation DNA sequencing data. Genome Res. *20*, 1297–1303.

45. Cingolani, P., Platts, A., Wang, L., Coon, M., Nguyen, T., Wang, L., Land, S.J., Lu, X., and Ruden, D.M. (2012). A program for annotating and predicting the effects of single nucleotide polymorphisms, SnpEff: SNPs in the genome of Drosophila melanogaster strain w1118; iso-2; iso-3. Fly (Austin) *6*, 80–92.

46. Robinson, J.T., Thorvaldsdóttir, H., Winckler, W., Guttman, M., Lander, E.S., Getz, G., and Mesirov, J.P. (2011). Integrative genomics viewer. Nat. Biotechnol. *29*, 24–26.

47. Campbell, C.D., Chapman, S.J., Cameron, C.M., Davidson, M.S., and Potts, J.M. (2003). A rapid microtiter plate method to measure carbon dioxide evolved from carbon substrate amendments so as to determine the physiological profiles of soil microbial communities by using whole soil. Appl. Environ. Microbiol. *69*, 3593–3599.

48. Bailey, S.F., Blanquart, F., Bataillon, T., and Kassen, R. (2017). What drives parallel evolution?: How population size and mutational variation contribute to repeated evolution. BioEssays *39*, 1–9.

STAR★METHODS

KEY RESOURCES TABLE

REAGENT or RESOURCE	SOURCE	IDENTIFIER
Bacterial and Virus Strains		
Stenotrophomonas sp. D12	Michael Brockhurst [28]	N/A
Bacillus sp. D26	Michael Brockhurst [28]	N/A
Paenibacillus sp. A8	Michael Brockhurst [28]	N/A
Microbacterium sp. D148	Michael Brockhurst [28]	N/A
Cellulomonas sp. D13	Michael Brockhurst [28]	N/A
Rhodococcus sp. E31	Michael Brockhurst [28]	N/A
Deposited Data		
Genome sequence data	European Nucleotide Archive	PRJEB36888
Experimental data	This paper	Data S1
Called sequence variant	This paper	Data S2
Software and Algorithms		
R version 3.5.1	https://www.r-project.org/	N/A
PRISM version 8.1.2	https://www.graphpad.com/scientific-software/prism/	N/A
Qiime version 1.9.1	http://qiime.org/ [36]	N/A
Canu	https://github.com/marbl/canu [37]	N/A
Circulator	https://github.com/sanger-pathogens/circlator [38]	N/A
Pilon	https://github.com/broadinstitute/pilon [39]	N/A
Prokka	https://github.com/tseemann/prokka [40]	N/A
KEGG automated annotation server	https://www.genome.jp/kegg/kaas/ [41]	N/A
Interpro	https://www.ebi.ac.uk/interpro/ [42]	N/A
SAMtools	https://github.com/samtools/ [43]	N/A
picard	https://broadinstitute.github.io/picard/ [44]	N/A
SNPeff	https://pcingola.github.io/SnpEff/ [45]	N/A
igv	http://software.broadinstitute.org/software/igv/ [46]	N/A

RESOURCE AVAILABILITY

Lead Contact

Further information and requests for resources should be directed to and will be fulfilled by the lead contact, Michael Brockhurst (michael.brockhurst@manchester.ac.uk).

Materials availability

The ancestral bacterial isolates used in this study are available upon request.

Data availability

All experimental data is provided in the supplemental data file Data S1. Genomic data is available at the European Nucleotide Archive, accession PRJEB36888, and in the supplemental data file Data S2.

EXPERIMENTAL MODEL AND SUBJECT DETAILS

Bacterial strains and culture conditions

All isolates used in this experiment were previously isolated from wheat straw compost enrichment cultures [28]. Six isolates were used in this study: *Stenotrophomonas* sp. D12, *Bacillus* sp. D26, *Paenibacillus* sp. A8, *Microbacterium* sp. D148, *Cellulomonas* sp. D13, *Rhodococcus* sp. E31. Overnight cultures of isolates were grown in nutrient broth at 30°C shaken orbitally at 150 rpm for 24 h. Wheat straw microcosms comprised 6 mL M9 solution supplemented with 60 mg wheat straw as the sole carbon source. Wheat straw microcosm cultures were incubated at 30°C shaken orbitally 150 rpm for 6 days.

METHOD DETAILS

Selection experiment

All isolates used in this experiment were previously isolated from wheat straw compost enrichment cultures [28]. *Stenotrophomonas* sp. D12 was used as the focal species; this isolate was naturally highly resistant to kanamycin. Five additional strains from the Bacillus, Paenibacillus, Microbacterium, Cellulomonas and Rhodococcus genera were chosen that exhibited varying metabolic traits and sensitivity to kanamycin allowing us to recover the focal species from polyculture by selective plating on media supplemented with kanamycin. We established 3 treatments: monocultures (MC) where the focal species evolved alone, reset polycultures (RP) where the focal species evolved in a community that was held constant over time, and dynamic polycultures (DP) where the focal species evolved in a community wherein all species could make ecological and evolutionary responses to selection. To establish the experimental lines, we picked twelve independent colonies of *Stenotrophomonas* that had been previously streaked onto a nutrient agar plate. These were named clones A-L.

All isolates were grown overnight in nutrient broth at 30°C, 150 rpm then diluted to approximately 10^6 cells/mL. These cultures were used to inoculate wheat straw microcosms, comprising 6 mL M9 solution with 60 mg wheat straw. A schematic diagram of the growth cycles used in each treatment is provided in Figure S4. Monocultures (MC) were inoculated with 60 μL of the focal species and polycultures were inoculated with 10 μL of each species. Using the independent clones, A-L, twelve replicate MC populations and twelve replicate polyculture communities were established to yield 24 wheat straw microcosms which were incubated at 30°C, 150 rpm for 6 days. Half of the polyculture replicates were propagated as dynamic polycultures (wherein species sorting was permitted; DP; clones A-F) whereas the other half of the replicates were propagated as reset polycultures (wherein species sorting was not permitted; RP; clones G-L), as follows: At each serial transfer, cultures were diluted to 1:100,000 in M9 solution and 100 μl was spread onto nutrient agar plates (DP and MC treatments) or nutrient agar plates containing 50 μg/mL kanamycin (RP treatment) to isolate only the *Stenotrophomonas* sp. D12 population, giving approximately 400-800 colonies per plate. Plates were incubated overnight at 30°C then 1 mL M9 solution was added to each plate and the colonies were disrupted using a spreader. 100 μL of this cell suspension was transferred to a microplate and cells were pelleted by centrifugation, then washed and suspended in M9 solution. For the DP and MC treatments, wheat straw microcosms were inoculated with 60 μl of these cell suspensions. For the RP treatment, wheat straw microcosms were inoculated with 10 μl of the cell suspension and 10 μl of each additional species grown overnight in nutrient broth from ancestral glycerol stocks and diluted to 10^6 cells/mL. The experiment was run for 16 growth cycles. Samples of the *Stenotrophomonas* sp. evolving population from each replicate were stored as cryogenic glycerol stocks at −80°C after every fourth growth cycle. Note that we did not perform additional controls for the effect of kanamycin selection and cannot rule out that kanamycin resistance frequency may have declined in treatments that were not regularly plated onto kanamycin plates.

Resource use assays

To quantify the metabolic traits of the ancestral and evolved *Stenotrophomonas* sp. D12 populations, growth assays were performed on several polysaccharides present in lignocellulose as previously described [28]. Hemicellulose substrates included xylan (Sigma-Aldrich), arabinoxylan (P-WAXYL, Megazyme) and galactomannan (P-GALML, Megazyme); cellulose substrates included β-glucan (P-BGBL, Megazyme) and Whatman filter paper; additional substrates included pectin (Sigma-Aldrich) and Kraft lignin (Sigma-Aldrich). Ancestral *Stenotrophomonas* sp. D12 was grown from glycerol stocks overnight in nutrient broth at 30°C, 150 rpm. Cultures were harvested by centrifugation, washed and suspended in M9 solution and left at room temperature for 2 h to metabolise any remaining nutrients. Evolved *Stenotrophomonas* sp. D12 populations from transfers 4, 8, 12 and 16 were isolated by diluting communities to 10^{-5} and plating 100 μL onto nutrient agar plates with 50 μg/mL kanamycin. Plates were incubated at 30°C for 24 h then 1 mL M9 media was added to plates and colonies were disrupted with a spreader. 100 μL of culture was added to 900 μL M9 minimal media and cells were harvested by centrifugation, washed and suspended in 1ml M9 minimal media then left for 2 h at room temperature to metabolise remaining nutrients. All cultures were standardized to an OD_{600} of 0.1 and 5 μL of culture was used to inoculate 495 μl of M9 solution with 0.2% (w/v) of each carbon source or one 6mm sterile filter paper disc in 96-well deepwell plates. Each plate

CellPress
OPEN ACCESS

contained an uninoculated blank well containing only 500 μl of M9 solution against which optical density measurements of the active wells were normalized. Cultures were grown for 5 days at 30°C and the MicroResp system was used to measure culture respiration as previously described [47].

Autonomous and competitive growth assays

To measure the autonomous growth of ancestral and end-point evolved *Stenotrophomonas* sp. D12 populations on wheat straw we inoculated wheat straw microcosms with 10 μL of an overnight culture. Each population was grown for 6 days at 30°C, 150 rpm. Starting and final population densities of samples were quantified by plating a serial dilution onto nutrient agar after 0 and 6 days of incubation. To measure competitive growth of ancestral and end-point evolved *Stenotrophomonas* sp. D12 populations on wheat straw in the presence of the ancestral community we inoculated wheat straw microcosms with 10 μL of the community of additional species (10^6 *cf.*u/mL) grown from ancestral glycerol stocks and 10 μL of each evolved focal species population or the ancestral genotype. Cultures were grown for 6 days at 30°C, 150rpm. Starting and final population densities of samples were quantified by plating a serial dilution onto nutrient agar supplemented with 50 μg/mL kanamycin after 0 and 6 days of incubation. Autonomous and competitive growth rates were calculated as the Malthusian parameter.

Genome sequencing

We first obtained the complete closed genome sequence of the *Stenotrophomonas* sp. D12 ancestral strain. A single colony of the ancestral *Stenotrophomonas* sp. D12 was resuspended in nutrient broth and grown overnight at 30°C, 150 rpm. Cells were harvested, and genomic DNA was extracted using QIAGEN Genomic Tips 20G (QIAGEN, Germany). Long-read sequencing using the PacBio Sequel System (Pacific Biosciences; performed by NERC Biomolecular Analysis Facility at the University of Sheffield), produced 377,905 reads with an average length of 5,597 bp. Additional short read sequencing was performed using the Illumina MiSeq platform by MicrobesNG (University of Birmingham, www.microbesng.com), produced 337,653 2x250 paired-end reads with a median insert size of 529 bp representing 59x genome coverage. To identify mutational changes in evolved clones, a single clone of *Stenotrophomonas* sp. D12 from each of the 24 evolved populations was sequenced using the Illumina Miseq platform by MicrobesNG (University of Birmingham, www.microbesng.com).

Amplicon sequencing

To determine the relative abundance of each species in each of the DP communities over time, we isolated total genomic DNA at every fourth transfer using the QIAGEN DNeasy Blood and Tissue kit following the manufacturer's protocol for Gram-positive bacteria. 16S-EZ amplicon sequencing of the V3 and V4 hypervariable regions of the 16S rRNA gene was performed by Genewiz (New Jersey, USA). Briefly, the V3/V4 regions were amplified by PCR and sequenced using the Illumina platform with 2x250 bp paired-end reads.

QUANTIFICATION AND STATISTICAL ANALYSIS

Statistical analysis

All statistical analyses were performed using R (version 3.5.1) or PRISM (version 8.1.2.). Change in resource use was compared between treatments using a repeated-measures linear mixed effects model (nlme R package). Evolutionary paths among all treatments through time were determined and analyzed by Phenotypic Trajectory Analysis (PTA [31]). PTA is initiated with a model evaluating whether the effect of time on the multivariate substrate use response variable varies by treatment. The model is fit by the "Randomization of Residuals in a Permutation Procedure" which is used to construct ANOVA tables that are functionally similar to a non-parametric likelihood ratio test. Following fitting this model (essentially a MANOVA), the trajectory analysis constructs distance and vector based pairwise comparisons of trajectories in the full multivariate space, resulting in inference about path length, shape and direction of evolutionary change, through time, for each treatment (function 'trajectory.analysis' from the geomorph R package). Visualizing the trajectories (centroids, path lengths, path direction) is made possible by estimating principal components of the fitted values from the model and projecting the data onto them [31]. Divergence of metabolic phenotype and community structure was calculated using Bray-Curtis dissimilarity, and their relationship was analyzed using linear regression. Numbers of mutations between treatments were compared using Welch's anova. The similarity in mutational profiles between all possible pairs of sequenced clones was calculated using the Jaccard Index (Jaccard Index = number of loci targeted in common / total number of loci targeted [48];), to create a similarity matrix (Figure S3). The degree of parallel evolution between and within treatments was then analyzed as the effect of treatment comparison on the Jaccard index (i.e., whether pair of clones from same or different named treatments), using a permutational ANOVA (10,000 permutations).

Genome sequencing bioinformatics

PacBio reads were assembled *de novo* using Canu with default settings [37]. Following correction and trimming, 334,634 reads with an average length of 5,148 bp representing 370x genome coverage were assembled into two contigs. These contigs were combined using Circlator with default settings [38], then polished with Illumina Miseq reads using Pilon [39]. The resulting genome, which totalled 4,659,921bp, was then annotated using Prokka [40], with additional functional annotation using the KEGG automated annotation server (KAAS) [41] and InterPro [42].

To identify mutational changes in evolved clones, paired reads were aligned to the annotated ancestral genome sequence using Burrows-Wheeler Aligner in SAMtools [43] and duplicate reads were removed using the MarkDuplicates function from picard (https://broadinstitute.github.io/picard/). Variants were called using GATK Haplotype Caller [44] and annotated using SNPeff [45]. Called variants were then filtered in R (version 3.5.1) to remove low quality calls with either low coverage (< 10 reads per bp) or low frequency of the alternative allele (< 80% of reads with alternative). Variant calling was also performed on the ancestral short-read Illumina data which was used to polish the PacBio reference genome in order to identify any variants which were present in the ancestor and missed by the polishing step; subsequently, any variants present in the ancestral and evolved sequences were discounted from further analysis. All variants were validated visually using the alignment viewer igv [46].

Amplicon sequencing bioinformatics

Raw reads were optimized by assembling read pairs and removing undetermined bases and primer and adaptor sequences. Chimeric sequences were removed. Qiime (version 1.9.1) [36] was used to assemble reads into operational taxonomic unit (OTU) clusters (similarity = 97%), which were identified and the relative abundance of each OTU was calculated. OTUs were annotated using the Greengenes comparison database in Qiime. Annotated OTUs matched the six expected genera, unclassified OTUs were removed from further analysis.

Current Biology, Volume *30*

Supplemental Information

Eco-evolutionary Dynamics Set the Tempo and Trajectory of Metabolic Evolution in Multispecies Communities

Rachael Evans, Andrew P. Beckerman, Rosanna C.T. Wright, Simon McQueen-Mason, Neil C. Bruce, and Michael A. Brockhurst

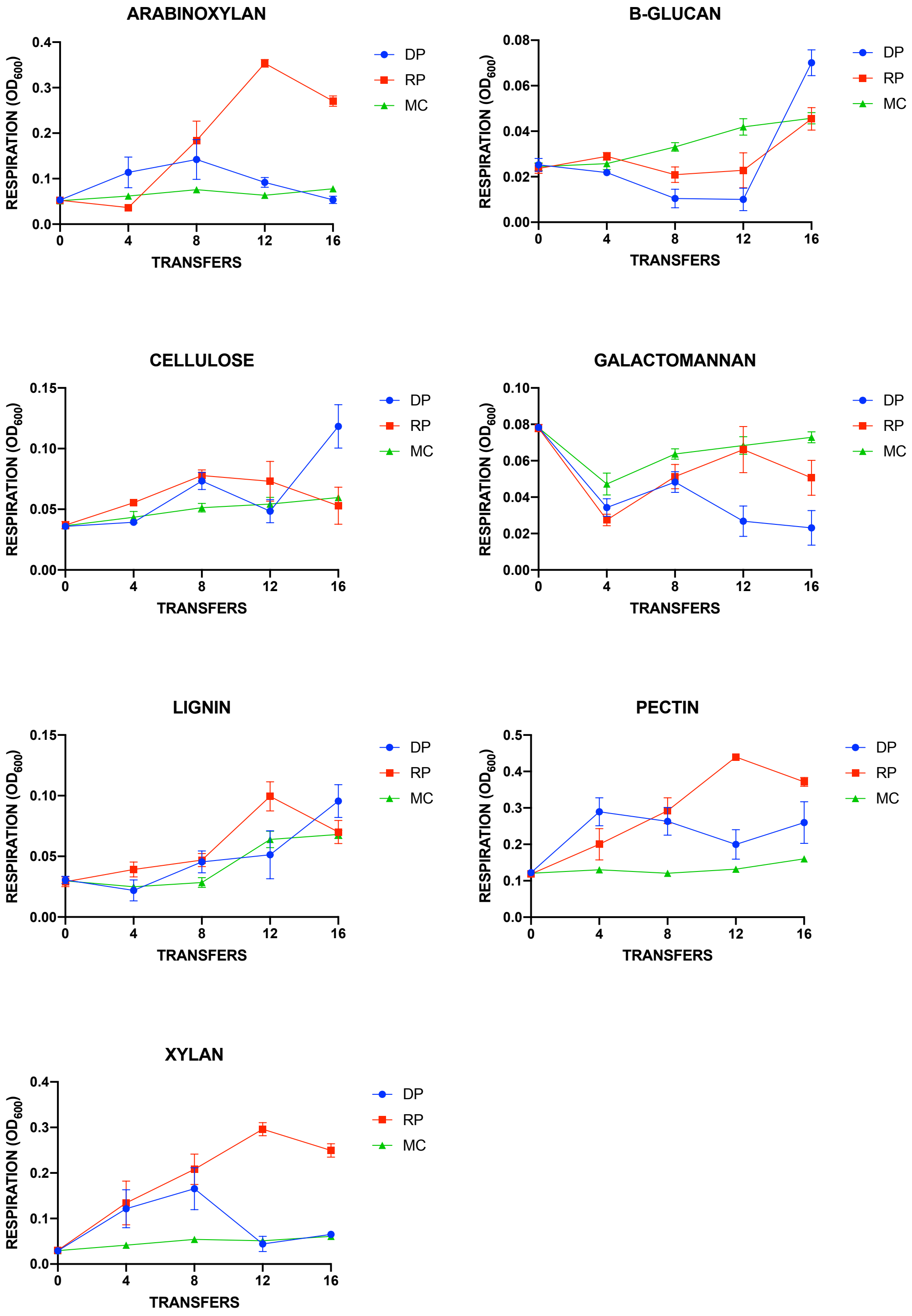

Figure S1 | Carbon substrate use over time. Related to Figure 1. Dots indicate mean ± s.e.m. respiration on a carbon substrate measured as optical density absorbance at 600 nm wavelength for *Stenotrophomonas* sp. populations from the evolution treatments [monoculture (MC; green),

reset polyculture (RP; red), dynamic polyculture (DP; blue)] over time denoted as the number of serial transfers. Panels show utilisation of different carbon substrates as indicated by the title of each panel.

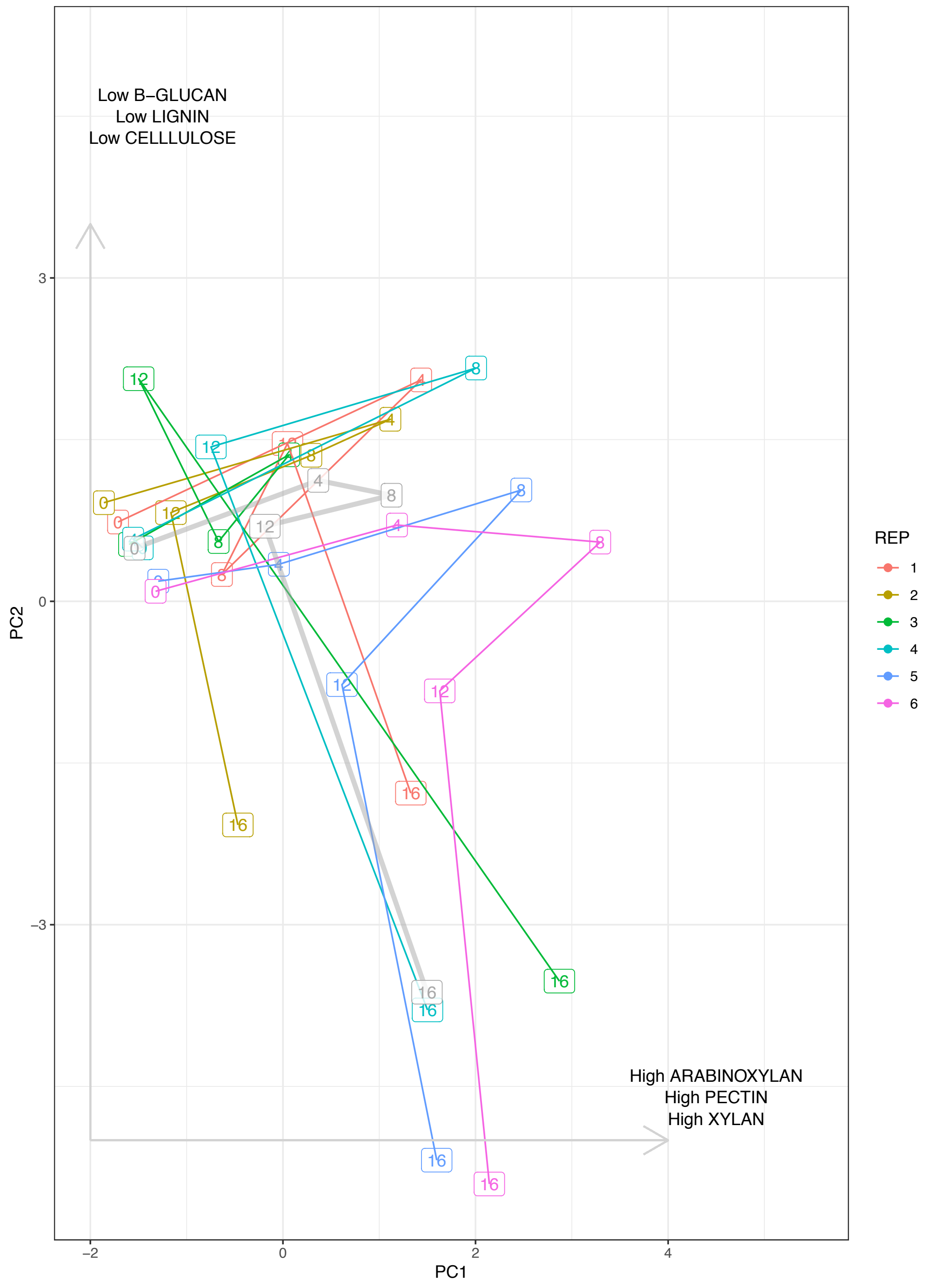

Figure S2 | Individual replicate trajectories of *Stenotrophomonas* sp. metabolic phenotype evolution in the Dynamic Polyculture treatment. Related to Figure 1. Ordination plot from a Principal Components Analysis, lines show evolutionary trajectories for the individual replicate from the dynamic polyculture treatment. Colours denote replicate as detailed in the graphical key.

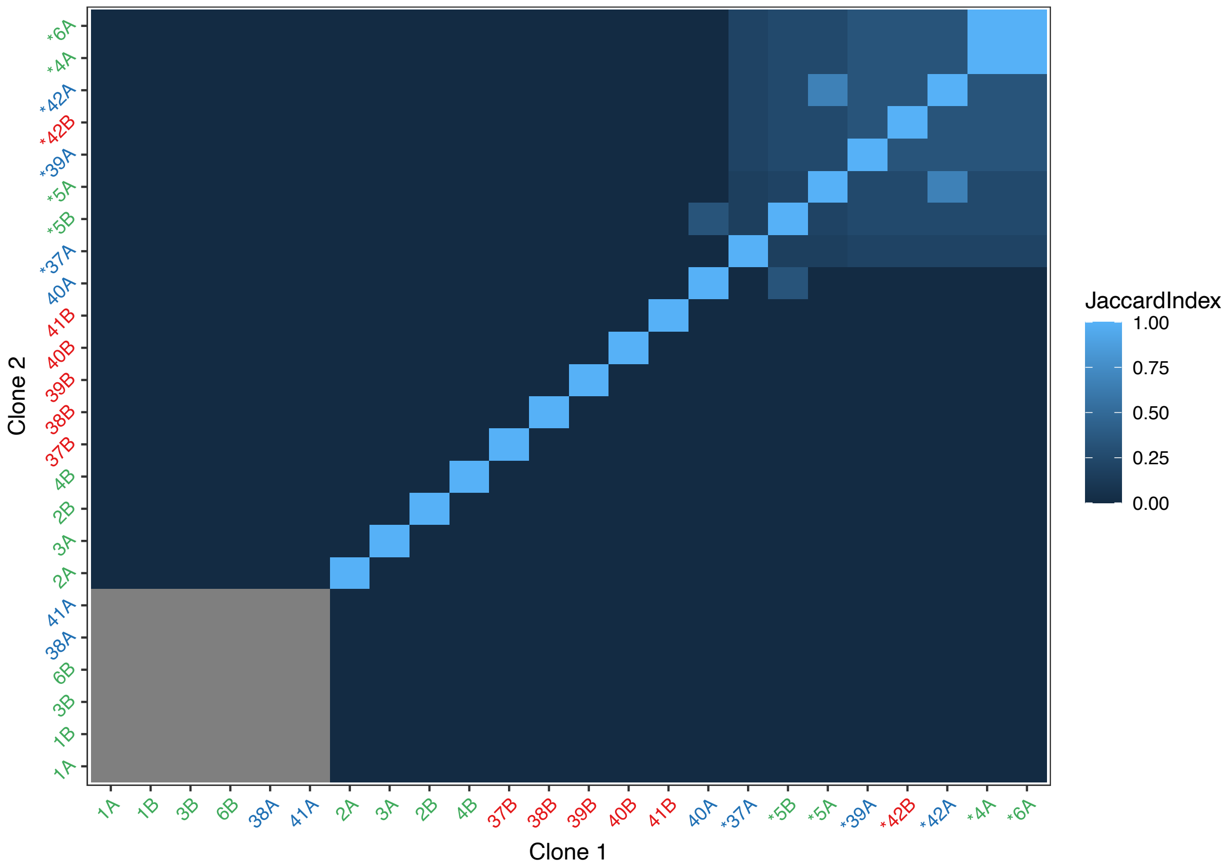

Figure S3 | Pairwise genetic similarity of all sequenced clones. Related to Figure 4. Similarity matrix showing Jaccard index values for all pairwise combinations of sequenced clones. Jaccard index was calculated as number of loci targeted in common divided by the total number of loci targeted, such that 0 (dark blue) indicates no loci in common, and 1 (light blue) indicates identical mutational profiles. Grey indicates that sequenced clones had no identifiable mutations. Clone name labels are coloured according to their treatment: green, monoculture (MC); red, reset polyculture (RP); blue, dynamic polyculture (DP). Asterisks denote clones carrying a mutation in the *tctE* gene. Each clone name relates to a circle plotted in Figure 4, as follows: from the innermost to the outermost circle in Figure 4, clones are arranged from 1A to 6A then 1B to 6B for the monoculture treatment (green), from 37A to 42A for the dynamic polyculture treatment (blue), and from 37B to 42B for the reset polyculture treatment (red).

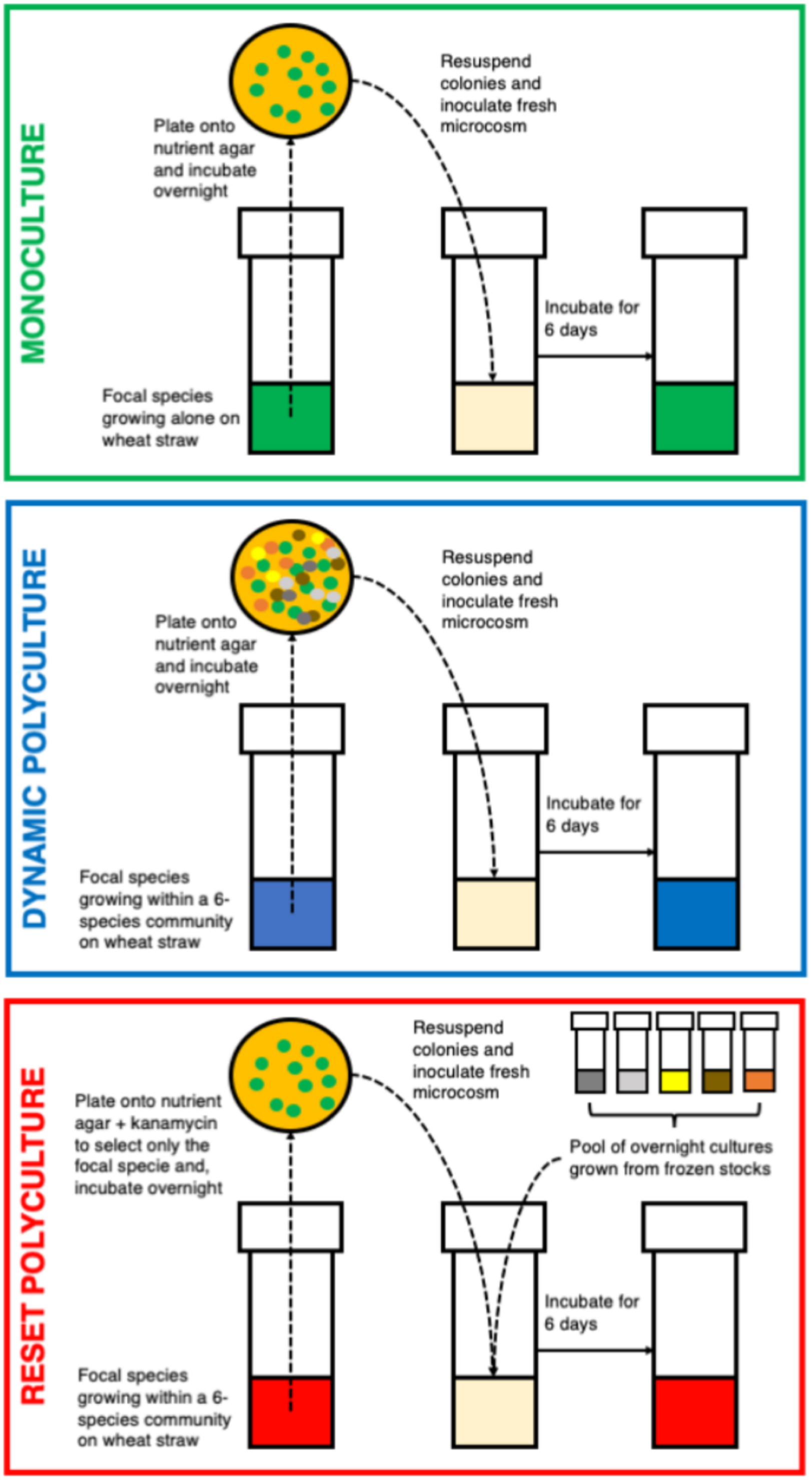

Figure S4 | Schematic diagram of the growth cycles used in each treatment. Related to the STAR Methods.
